# The effects of aerobic exercise on sleep quality in older adults with sleep problems: a systematic review and meta-analysis of randomized controlled trials

**DOI:** 10.3389/fpsyg.2026.1743800

**Published:** 2026-02-24

**Authors:** Hongyi Liao, Haoming Yan, Manhui Xia, Huihong Den

**Affiliations:** 1 School of Physical Education, Kashi University, Kashi, China; 2 School of Physical Education, Chengdu Sport University, Chengdu, China; 3 School of Physical Education and Health, Guizhou Minzu University, Guiyang, China; 4 Guangdong University of Foreign Studies South China Business College, Guangzhou, China

**Keywords:** aerobic exercise, meta-analysis, older adults, sleep problem, sleep quality

## Abstract

**Objective:**

Sleep problems among older adults have long been a major concern in the field of public health. For this population, aerobic exercise is considered a more appropriate non-pharmacological approach to improving sleep quality. Nonetheless, there is a scarcity of concrete evidence focusing on older adults who have clinically recognized sleep problems. As a result, the objective of this research is to assess the impact of aerobic exercise on the sleep quality of older adults experiencing sleep problems.

**Methods:**

A thorough investigation was performed across five primary databases: PubMed, Embase, Cochrane Library, Web of Science, and EBSCO, aimed at identifying all pertinent studies released up until September 30, 2025. The search method specifically targeted randomized controlled trials (RCTs) that explored the relationship between aerobic exercise interventions and sleep quality outcomes among older adults experiencing sleep problems. To guarantee comprehensiveness, the search included all accessible records from the beginning of each database's establishment.

**Results:**

After systematic screening and comprehensive evaluation, a total of 12 studies were identified from 2,752 initial records that met the inclusion criteria for meta-analysis. All participants were older adults with sleep problems. Data analysis revealed that aerobic exercise had a significant positive effect on improving sleep quality in this population (SMD = −0.98, 95% CI: −1.36 to −0.60, *p* < 0.001). Subgroup analyses further indicated that various parameters of the exercise intervention—including weekly frequency, session duration, and total intervention period—had statistically significant positive effects on sleep quality.

**Conclusion:**

The results of this study indicate that exercise therapy can significantly improve sleep quality in older adults with sleep disorders. In terms of intervention frequency, programs conducted more than three times per week achieved the best outcomes. Moreover, the findings suggest that exercise sessions lasting 60 min or longer were more effective than shorter sessions, while a 12-week intervention period produced the most favorable clinical benefits. Overall, these results provide a solid scientific basis for developing precise exercise therapy programs and health management strategies aimed at optimizing sleep quality among older adults. When designing exercise prescriptions, it is essential to fully consider individual differences to ensure long-term adherence and safety.

**Registration:**

PROSPERO, Registration Number: CRD420251156878.

## Introduction

Sleep problems are highly prevalent among older adults and have become a major public health issue worldwide (Ding et al., 2023). Epidemiological studies indicate that more than 50% of older adults experience some form of sleep disturbance, including difficulties with sleep onset, reduced sleep duration, frequent awakenings, or poor sleep efficiency (Dasdemir Ilkhan and Celikhisar, 2021; Gordon et al., 2022). These issues are strongly linked to numerous negative results, such as cognitive deterioration, depression, cardiovascular illnesses, weakened immune response, and a markedly diminished quality of life (Chen et al., 2023). With the global trend of population aging intensifying, improving sleep quality in older adults has emerged as an urgent healthcare priority.

Pharmacological treatments, such as sedative-hypnotic medications, are commonly prescribed to manage and improve sleep problems in older adults (Godfrey et al., 2022). However, their use is often limited by side effects, drug tolerance, dependence, and potential interactions with medications for comorbid conditions (Anghel et al., 2022). Moreover, due to the generally reduced physiological resilience of older adults compared with younger and middle-aged adults, such treatments may lead to more pronounced and severe adverse reactions (Tuft et al., 2023). Among these, exercise interventions have been widely recognized as an effective alternative for improving sleep quality (Park et al., 2021). Various targeted exercise interventions have been broadly applied in sleep management programs for different populations (Bugday et al., 2025; Pehlevan and Sevgin, 2024; Koçak and Sevgin, 2025). Aerobic exercise has attracted increasing attention due to its accessibility, adaptability, and multifaceted health benefits, and it is particularly suitable for older adults experiencing declines in physical strength (Miyazaki et al., 2021). Research indicates that engaging in aerobic activities can significantly enhance the quality of sleep and diminish fatigue among adults (Arslan and Sevgin, 2024). However, although several RCTs have examined the effects of aerobic exercise on sleep outcomes in older adults, their findings remain inconsistent (Zhou et al., 2022; Marupuru et al., 2022; Brupbacher et al., 2021). Variations in study design, intervention type, exercise intensity, duration, and outcome measures may contribute to these discrepancies.

Although several previous systematic reviews and meta-analyses have examined the relationship between physical activity or exercise and sleep outcomes, most have focused on mixed-age populations, heterogeneous exercise modalities, or general sleep outcomes without targeting older adults with clinically relevant sleep problems (Rubio-Valles and Ramos-Jimenez, 2025; Zhou et al., 2025; Korkutata et al., 2025). Moreover, limited attention has been paid to identifying the optimal characteristics of aerobic exercise interventions. To our knowledge, no meta-analysis has specifically synthesized randomized controlled trials of aerobic exercise interventions in older adults with sleep problems while simultaneously examining key intervention parameters such as exercise frequency, session duration, and total intervention length. Therefore, a rigorous and up-to-date review is needed to clarify the effects of aerobic exercise on sleep quality in this population. To fill this gap, this systematic review and meta-analysis aimed to assess how aerobic exercise affects sleep quality in older adults experiencing sleep problems, utilizing data from randomized controlled trials. By integrating results from various studies, this research intends to offer solid evidence that can enhance clinical practices and aid in creating effective non-pharmacological strategies to improve sleep health in older individuals.

## Methods

### Registration

In alignment with the pertinent guidelines outlined by the Preferred Reporting Items for Systematic Reviews and Meta-Analyses (PRISMA) (Page et al., 2022), a standardized implementation procedure has been carried out for this study. Furthermore, the research is registered with the international prospective register of systematic reviews, PROSPERO, under the unique identification number CRD420251156878. The study protocol's design and execution were rigorously compliant with PRISMA guidelines, which helped guarantee the research process's standardization and the reliability of the findings.

### Search strategy

In this study, a systematic search was conducted across five authoritative databases, including Web of Science, EBSCO, PubMed, Embase, and the Cochrane Library, to identify all relevant literature published up to September 30, 2025. This study employed the following terms and operators: (“aerobic exercise^*^”[Title/Abstract] OR “aerobic training”[Title/Abstract] OR “endurance exercise^*^”[Title/Abstract] OR “cardiovascular exercise^*^”[Title/Abstract] OR “swimming”[Title/Abstract] OR “bicycle”[Title/Abstract] OR “walking”[Title/Abstract] OR “running”[Title/Abstract] OR “tai chi”[Title/Abstract]) AND (“Aged”[MeSH Terms] OR “Older adults”[Title/Abstract]) AND (“Sleep”[MeSH Terms] OR “Sleep Quality”[MeSH Terms] OR (“sleep disturbance^*^”[Title/Abstract] OR “sleep maintenance”[Title/Abstract] OR “sleep disorder^*^”[Title/Abstract] OR “sleep problem^*^”[Title/Abstract] OR “sleeplessness”[Title/Abstract] OR “sleep duration^*^”[Title/Abstract] OR “sleep health”[Title/Abstract])) AND (“randomized controlled trial”[Title/Abstract] OR “randomized”[Title/Abstract] OR “placebo”[Title/Abstract]). In order to achieve thoroughness, we also examined the reference lists of every article obtained to discover any further qualifying studies. The comprehensive search approach is outlined in the Supplementary materials.

### Eligibility criteria

For the purpose of this review, “sleep problems” were operationally defined as the presence of clinically relevant sleep disturbances identified by validated subjective or objective sleep assessment tools. This study included only English-language articles without restrictions on publication date, and all eligible studies met the PICOS criteria. The inclusion criteria for participants were as follows: (1) older adults aged over 60 years with a confirmed diagnosis of sleep problems; (2) Aerobic exercise serves as the intervention, utilizing frequently employed methods such as walking, Tai Chi, and Baduanjin, among others (Wang et al., 2022; Li et al., 2024); (3) control group participants maintaining their usual lifestyle, not engaging in exercise, or receiving other standard treatments; and (4) availability of data records on changes in sleep quality.

The exclusion criteria were as follows: (1) participants with severe physical or mental illnesses, such as malignant tumors; (2) absence of a control group design; (3) lack of outcomes related to sleep quality; (4) incomplete data that could not be extracted, such as missing outcome measures, insufficient statistical detail; and (5) specific publication types like conference abstracts, retrospective analyses, monographs, correspondence, or patents.

### Study selection

The selection of studies was carried out following the PRISMA guidelines. Two reviewers, both authors of this manuscript with expertise in physical education and exercise science, conducted an independent screening of titles and abstracts, having prior training in systematic review methodologies. The same reviewers then evaluated full-text articles for eligibility according to the established inclusion and exclusion criteria. In cases where disagreements arose during the screening, discussions were held to reach a resolution. If a consensus could not be achieved, a third author was brought in to resolve the issue and make the final determination.

### Data extraction

Three reviewers conducted the data extraction independently. Any conflicts that emerged during this process were addressed through a consensus mechanism involving the entire team. The variables extracted encompassed several essential components, such as the primary author of the study, the year it was published, the overall sample size, the age distribution of participants, specific variations in interventions between the control and experimental groups, dosage specifications of the intervention, and diverse assessment data indicating sleep quality. In addition to basic study characteristics and outcome data, outcome measures were extracted based on participants who completed the intervention in each included trial. This per-protocol approach implicitly reflects a basic level of intervention adherence, as only participants who completed the exercise programs were included in the synthesis. When reported, we also attempted to extract adherence-related information (e.g., attendance or completion rates); however, such data were inconsistently reported across studies and could not be quantitatively synthesized.

### Study quality assessment

The risk of bias in the randomized controlled trials that were included was evaluated independently by two reviewers employing the Cochrane Risk of Bias tool. Each study underwent assessment across standard domains such as random sequence generation, allocation concealment, blinding, incomplete outcome data, selective reporting, and additional potential bias sources (Cumpston et al., 2019). Disagreements among the reviewers were settled through discussion. In cases where a consensus could not be reached, a third reviewer was brought in to provide the final decision. The overall certainty of the evidence was then evaluated using the GRADE approach (GRADE Working Group, 2004).

### Statistical analysis

The analysis of data was performed utilizing Review Manager 5.3. Given differences in measurement methods and units, such as the use of different instruments to assess sleep quality including the PSQI, CPSQI, and ISI, a random-effects model was applied in this meta-analysis (Borenstein et al., 2010). A 95% confidence interval (CI) was used to assess precision, while the I^2^ statistic (Viechtbauer and Cheung, 2010) was employed to evaluate heterogeneity among the studies. The threshold for statistical significance was set at a *p*-value of 0.05.

## Results

### Literature search results

**Figure 1:**
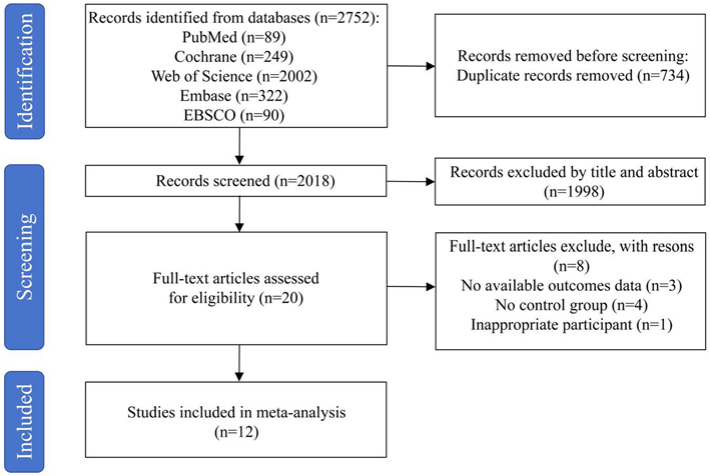
Flow diagram of literature selection process.

Through multi-database searches, a total of 2,752 records were initially identified. During the data preprocessing stage, 734 duplicate records were automatically detected and removed. Following that, a systematic screening of the titles and abstracts from the remaining studies was conducted, resulting in the exclusion of 1,998 records for lacking sufficient relevance. In the subsequent phase of full-text review, 8 articles were removed for not satisfying the predefined inclusion criteria. After this thorough selection procedure, 12 studies were identified as eligible and included in the meta-analysis. The flow diagram illustrates the comprehensive screening process and its outcomes (Figure 1).

### Basic characteristics of the included literature

The key features of the studies included are presented in Table 1. Each study was published within the timeframe of 2008–2025. The participants in all studies were older adults aged over 60 years. Aerobic exercise interventions in the experimental groups included Tai Chi, walking, aquatic exercise, running, Baduanjin, and aerobic dance. Across studies, intervention lengths spanned 4–24 weeks. Sessions generally lasted around 1 h and were most often scheduled three times per week. Sleep quality outcomes were measured through validated questionnaires, including the PSQI, CPSQI, and ISI.

**Table 1:** Characteristics of eligible studies.

**Included studies**	**Sample size (*N*)**	**Age of participants**	**Intervention**		**Diagnosis**	**Dose**	**Outcome measures**
			**E**	**C**			
Irwin et al. (2008), RCT	E = 59, C = 53	EG: 69.6 ± 6.2 CG: 70.2 ± 7.5	Tai Chi	Health education	Moderate sleep complaints	16 weeks, 40-min/session, 3 times/week	PSQI
Hosseini et al. (2011), RCT	E = 27, C = 29	EG: 68.7 ± 5.5 CG: 69.4 ± 5.3	Tai Chi	Non-exercise	Sleep disturbances	12 weeks, 25-min/session, 3 times/week	PSQI
Sharif et al. (2015), RCT	E = 30, C = 30	64.8 ± 5.2	Walking	Normal lifestyle	Sleep disorders	12 weeks, 60-min/session, 3 times/week	PSQI
Chen et al. (2016), RCT	E = 29, C = 34	EG: 65.2 ± 0.9 CG: 66.2 ± 1.2	Aquatic exercise	Normal lifestyle	Mild sleep impairment	8 weeks, 60-min/session, 2 times/week	Sleep actigraphy
Chan et al. (2016), RCT	E = 27, C = 25	EG: 79.4 ± 7.1 CG: 82.2 ± 6.7	Tai Chi	Routine activities	Sleep disorders	8 weeks, 60-min/session, 2 times/week	CPSQI
Abd El-Kader and Al-Jiffri (2019), RCT	E = 25, C = 25	EG: 65.0 ± 4.2 CG: 65.7 ± 3.9	Running and walking	Normal lifestyle	Difficulty falling asleep	24 weeks, 40-min/session, 3 times/week	PSG
Fan et al. (2020), RCT	E = 67, C = 72	EG: 70.3 ± 5.7 CG: 71.8 ± 6.7	Baduanjin	Normal lifestyle	Sleep disturbances	24 weeks, 45-min/session, 5 times/week	PSQI
Siu et al. (2021), RCT	E = 105, C = 110	EG: 66.5 ± 6.4 CG: 68.0 ± 8.2	Tai Chi	Usual care	chronic insomnia	12 weeks, 60-min/session, 3 times/week	PSQI, ISI
Chan et al. (2022), RCT	E = 23, C = 21	60–69	Tai Chi	Sleep hygiene education	Sleep disturbances	8 weeks, 120-min/session, 1 times/week	ISI
He et al. (2024), RCT	E = 13, C = 13	EG: 69.9 ± 5.4 CG: 69.6 ± 4.5	Tai Chi	Usual care	Sleep disturbances	4 weeks, 60-min/session, 3 times/week	ISI
Song et al. (2024), RCT	E = 45, C = 43	EG: 76.7 ± 6.0 CG: 75.2 ± 6.6	Aerobic dance	Health education	Poor sleep quality	16 weeks, 60-min/session, 3 times/week	PSQI
Liu et al. (2025), RCT	E = 51, C = 52	EG: 67.8 ± 4.5 CG: 68.0 ± 4.6	Tai Chi	rTMS	Sleep disorders	6 weeks, 60-min/session, 5 times/week	PSQI

E, experimental group; C, control group; PSQI, Pittsburgh Sleep Quality Index; rTMS, Repetitive Transcranial Magnetic Stimulation; CPSQI, Chinese Pittsburgh Sleep Quality Index; PSG, polysomnographic; ISI, insomnia severity index.

### Quality assessment

**Figure 2:**
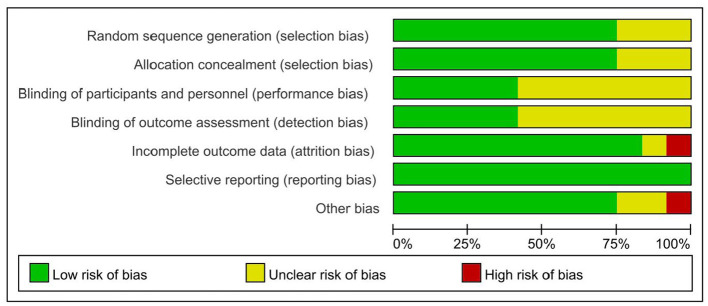
Overall overview graph of bias risk in included studies.

**Figure 3:**
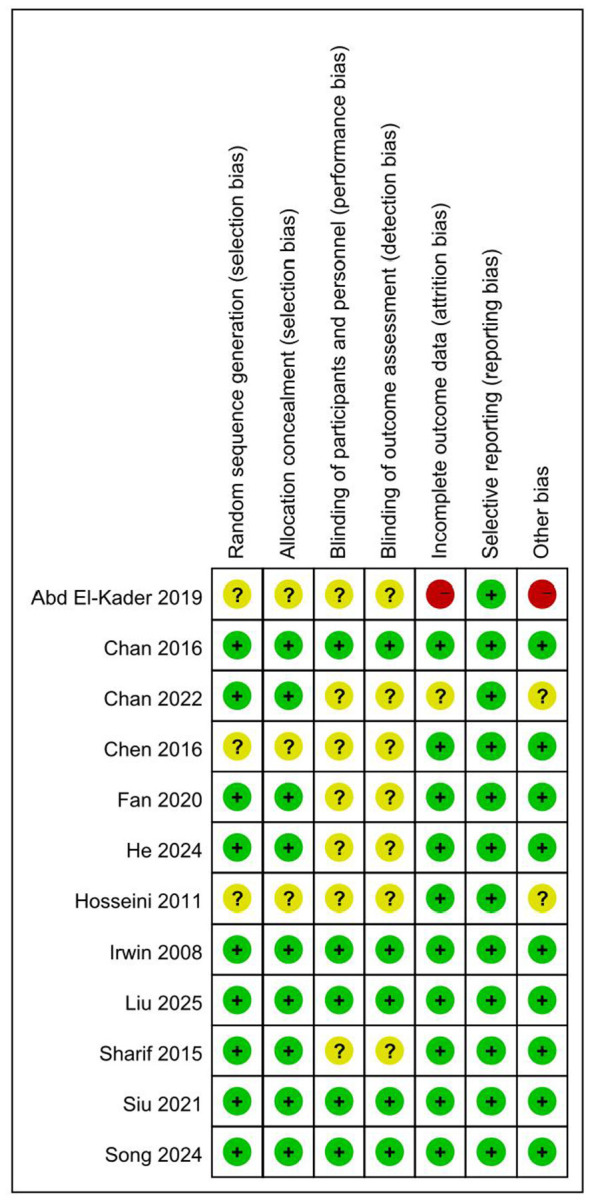
Risk of bias evaluation graph for the included literature.

Figures 2, 3 provide an extensive evaluation of bias risk, including both overall and individual analyses of study data. In the “Data Integrity” and “Other Potential Risks” categories, only a single study was assessed as having a high risk. The unique characteristics of exercise interventions create substantial operational hurdles for implementing blinding of participants, resulting in all studies being classified as low risk on this measure. In general, the clinical trials examined in this review reflect a strong methodological quality, offering a solid and reliable scientific foundation for the synthesis of the research. The assessment of evidence quality using the GRADE approach indicated that the overall certainty of the evidence was rated as “moderate”. Details of the evidence profile are provided in the Supplementary material.

### Meta-analysis

#### Overall effectiveness

**Figure 4:**
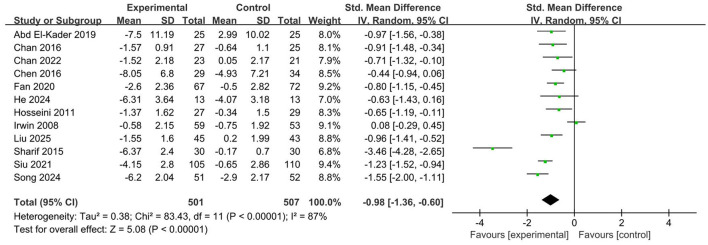
Forest plot of the overall effect of aerobic exercise intervention on sleep quality.

In comparison to the control group, there was a significant enhancement in sleep quality among older adults demographic as a result of aerobic exercise, accompanied by a marked decrease in sleep problems (SMD = −0.98, 95% CI: −1.36 to −0.60, *p* < 0.001) (Figure 4). Due to the considerable heterogeneity observed across the studies (*I*^2^ = 87%), the researchers performed a subgroup analysis to uncover possible sources of this heterogeneity and their contributing factors.

### Subgroup analysis

#### Intervention frequency

**Figure 5:**
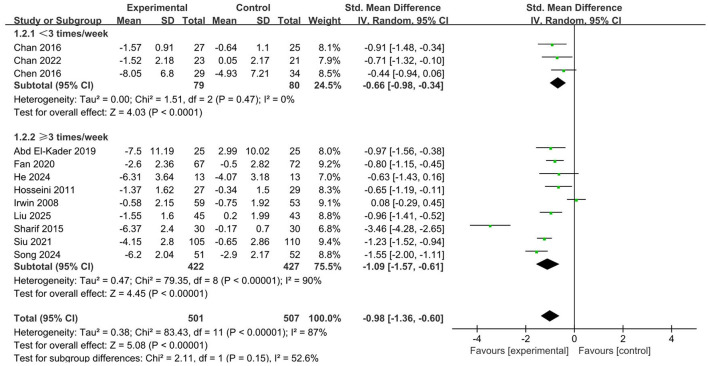
Forest plot of subgroup analysis by intervention frequency.

Both aerobic exercise interventions conducted less than three times a week (SMD = −0.66, 95% CI: −0.98 to −0.34, *p* < 0.001) and those performed three times or more weekly (SMD = −1.09, 95% CI: −1.57 to −0.61, *p* < 0.001) significantly enhanced sleep quality (Figure 5). Nonetheless, a greater frequency of these interventions proved to be more effective in enhancing sleep quality.

#### Duration of single intervention

**Figure 6:**
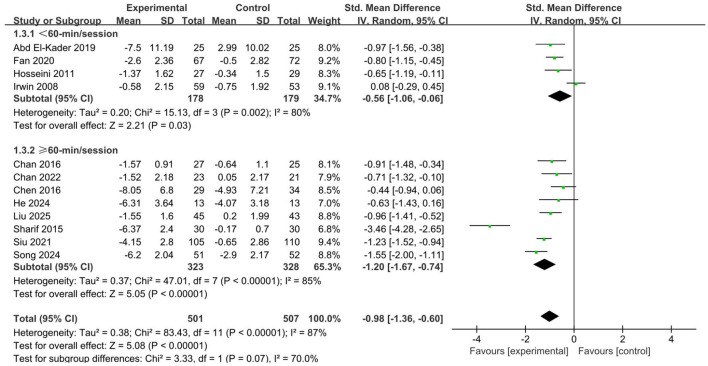
Forest plot of subgroup analysis by duration of single intervention.

Interventions involving aerobic exercise, whether lasting less than 60 min (SMD = −0.56, 95% CI: −0.98 to −0.34, *p* = 0.03) or 60 min and beyond (SMD = −1.20, 95% CI: −1.67 to −0.74, *p* < 0.001), demonstrated a significant positive impact on sleep quality (Figure 6). Furthermore, engaging in longer durations of single-session aerobic workouts offered a greater opportunity to improve sleep quality.

#### Total intervention period

**Figure 7:**
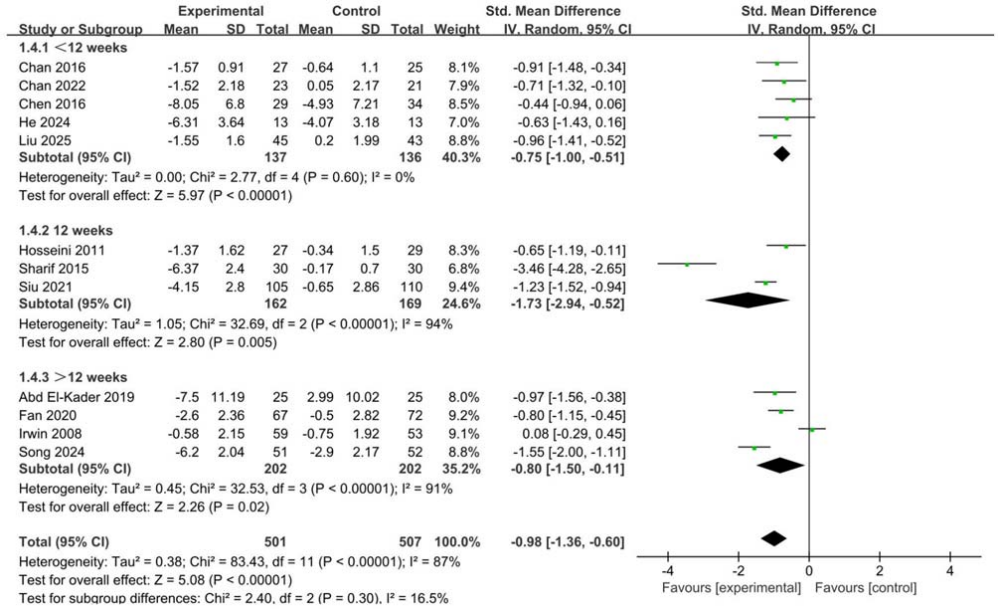
Forest plot of subgroup analysis by intervention cycle.

The results indicated that aerobic exercise interventions, whether lasting less than 12 weeks (SMD = −0.75, 95% CI: −1.00 to −0.51, *p* < 0.001), exactly 12 weeks (SMD = −1.73, 95% CI: −2.94 to −0.52, *p* = 0.005), or more than 12 weeks (SMD = −0.80, 95% CI: −1.50 to −0.11, *p* = 0.02), all produced significant improvements in sleep quality (Figure 7). When comparing intervention durations, those of moderate length demonstrated the most pronounced effects, while longer-term interventions ranked second in effectiveness.

### Sensitivity analysis and publication bias

**Figure 8:**
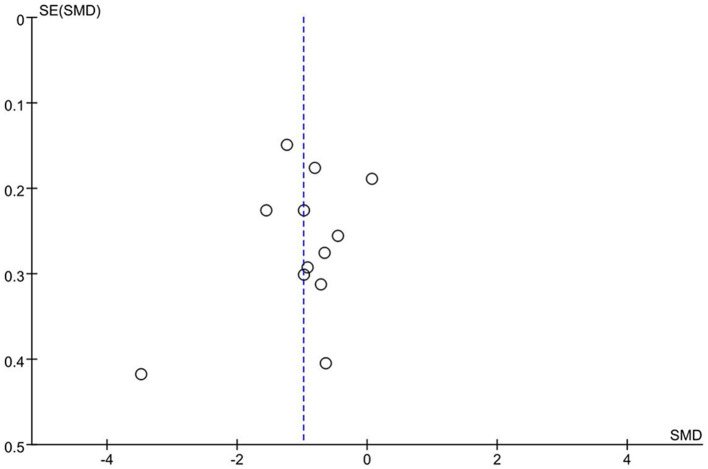
Publication bias analysis results.

By conducting a sensitivity analysis that involved the sequential removal of individual studies, the team discovered that none of the results from the analyses achieved a statistically significant level. To investigate possible publication bias, the research team utilized a funnel plot for detection purposes. The arrangement of the data points displayed a largely symmetrical nature, with only a single outlier observed. From this analysis, it may be concluded that there is a lack of significant publication bias in this area of research (see Figure 8).

## Discussion

This systematic review and meta-analysis investigated the effects of aerobic exercise on sleep outcomes in older adults with sleep disturbances. Based on randomized controlled trial (RCT) designs, strict inclusion criteria were applied to ensure that all participants exhibited varying degrees of sleep problems. The analysis demonstrated that aerobic exercise has significant therapeutic effects in this specific population, providing new empirical evidence for the field and confirming the broad applicability of aerobic exercise as an intervention to improve sleep problems.

Aerobic exercise can significantly improve sleep quality in older adults with sleep problems. This finding underscores the potential of aerobic exercise as a safe, cost-effective, and widely accessible non-pharmacological intervention for addressing sleep disturbances in older adults. Compared with pharmacological treatments, aerobic exercise carries a lower risk of adverse effects while simultaneously promoting overall physical and psychological health (Wong et al., 2023). The positive effects on sleep quality may be explained by multiple physiological and neurobiological mechanisms. From a physiological perspective, aerobic exercise enhances melatonin secretion, playing an important role in regulating circadian rhythms, thereby promoting a more consistent sleep–wake cycle and improving sleep efficiency (Kim et al., 2023). In addition, aerobic exercise promotes autonomic balance by reducing sympathetic activation and increasing parasympathetic activity, which helps relaxation and reduces nocturnal awakenings (Sato et al., 2021; Bommarito and Millar, 2024). Enhancements in cardiovascular performance and decreases in systemic inflammation brought about by aerobic exercise might signify crucial physiological mechanisms that contribute to improved sleep quality (Tayebi et al., 2025; Li et al., 2025). Prior research has shown a strong link between cardiovascular wellbeing and sufficient sleep, whereas ongoing inflammatory responses are acknowledged as possible contributors to sleep disruptions (Jaspan et al., 2024; Korostovtseva et al., 2021). Through the improvement of cardiorespiratory fitness, enhancement of blood flow, and reduction of inflammatory indicators, aerobic exercise not only fine-tunes the body's overall metabolic landscape but also creates better physiological circumstances for restorative sleep activities (Kozakova and Palombo, 2021). Secondly, aerobic exercise regulates key neurotransmitters by increasing γ-aminobutyric acid (GABA) activity and elevating serotonin and dopamine levels, which collectively promote deeper sleep and alleviate anxiety or depressive symptoms that often exacerbate insomnia in older adults (Demitrieva and Demitriev, 2025; Marrero-Cristobal et al., 2022). In addition, aerobic exercise enhances the expression of brain-derived neurotrophic factor (BDNF), thereby improving neuroplasticity and supporting the function of hypothalamic sleep centers (Lukkahatai et al., 2025). Finally, by attenuating the hyperactivity of the hypothalamic–pituitary–adrenal (HPA) axis and reducing cortisol secretion, aerobic exercise mitigates stress-related arousal, further facilitating restorative sleep (Park et al., 2023; Udi, 2025). It is important to emphasize that these potential mechanisms are based on previous experimental and observational studies and were not directly measured in the included trials; thus, they should be interpreted with appropriate caution.

Subgroup analysis based on intervention frequency indicated that both lower-frequency and higher-frequency aerobic exercise interventions significantly improved sleep quality, with higher-frequency programs demonstrating more pronounced effects. This outcome may be explained by behavioral and psychological factors rather than physiological mechanisms alone. More frequent exercise helps older adults establish a more stable daily routine and cultivate more persistent exercise habits, thereby promoting the development of healthy sleep schedules and enhancing the regularity of the sleep–wake cycle (Solis-Navarro et al., 2023; Ahn and Kim, 2023). In addition, exercising more frequently may improve psychological well-being by increasing opportunities for social interaction, strengthening self-efficacy, and fostering a greater sense of achievement, all of which contribute to alleviating anxiety and improving subjective sleep quality (Toros et al., 2023; Yao et al., 2021). From a practical perspective, higher-frequency interventions may also increase adherence by integrating exercise into daily life, turning it into a habitual behavior rather than an occasional activity (Harris-Love et al., 2021). However, it should be acknowledged that very high frequencies might challenge feasibility and sustainability for some older adults, especially those with physical limitations or comorbid conditions. Therefore, designing exercise programs that are frequent enough to generate behavioral reinforcement, but also realistic and adaptable to individual lifestyles, remains a key consideration for maximizing long-term benefits.

The results indicated a significant association between exercise duration and improvements in sleep quality. Comparative analyses showed that both shorter sessions of less than 60 min and longer sessions exceeding 60 min effectively enhanced sleep outcomes. However, further comparisons revealed that longer-duration exercise produced superior effects in promoting sleep. This difference may be attributed to multiple physiological responses induced by extended exercise duration (Johnson, 2023). Prolonged exercise leads to higher levels of energy metabolism and elicits deeper relaxation responses, and these combined effects ultimately contribute to more restorative and deeper sleep states (Korkutata et al., 2025). Moreover, increasing exercise duration not only helps enhance the self-efficacy and sense of accomplishment of older adults but also reinforces positive emotional feedback and self-motivation through the enjoyment experienced when completing exercise tasks (Xie et al., 2025; Easom, 2003). These pleasurable experiences promote long-term adherence to healthy lifestyle behaviors. Consistent engagement in exercise assists older adults in establishing stable daily routines and facilitates positive physiological cycles conducive to sleep, allowing both the body and mind to gradually adapt to a more regular lifestyle (Buman and King, 2010). Such long-term behavioral reinforcement plays a crucial role in improving sleep quality. Additionally, longer exercise sessions often provide greater opportunities for social interaction, such as group activities or community-based fitness programs. These social experiences can help alleviate loneliness, enhance emotional stability, and indirectly improve subjective sleep perceptions (Dahlberg et al., 2022; Liu et al., 2024). The combined increase in psychological satisfaction and social support further contributes to a higher quality of life and healthier sleep architecture (Zengin Alpozgen et al., 2022). Nevertheless, while extending exercise duration may offer additional benefits, it is essential to balance effectiveness with feasibility. For older adults with limited physical capacity or chronic diseases, excessively prolonged exercise may lead to fatigue accumulation or reduced adherence (Fragala, 2015). Therefore, designing individualized and progressive exercise prescriptions is critical to ensure that exercise maintains sufficient intervention intensity while remaining safe and sustainable.

Analysis of subgroups pertaining to the overall duration of interventions indicated that a 12-week aerobic exercise regimen resulted in the greatest enhancements in sleep quality. This was followed by interventions exceeding 12 weeks in length, with those lasting less than 12 weeks showing the least improvement. Overall, all three durations demonstrated significant positive effects. Although each duration exhibited beneficial outcomes, the 12-week intervention period appeared to achieve the optimal balance between the degree of exercise adaptation and participant adherence. This duration is sufficient to induce meaningful behavioral and psychological changes while maintaining feasibility and motivation among older adults. Interventions lasting more than 12 weeks may produce sustained positive effects; however, excessively long intervention periods can impose considerable physical and psychological stress on older adults. Given the decline in physiological function and reduced recovery capacity associated with aging, continuous exercise loads may lead to the accumulation of chronic fatigue (Eldadah, 2010). This persistent physical strain can result in discomfort, muscle soreness, or reduced motivation to exercise, thereby diminishing exercise tolerance and participation enthusiasm (Egerton et al., 2016; Morrison et al., 2016). In addition, prolonged interventions may induce “training fatigue” on a psychological level, characterized by decreased motivation, reduced interest, or resistance toward the exercise program. Without timely emotional regulation or external support, these negative effects may weaken the overall efficacy of the intervention or even offset the previously achieved benefits (Ament and Verkerke, 2009). In contrast, shorter intervention strategies (< 12 weeks) may have relatively limited long-term effects on sleep improvement, but their feasibility and therapeutic value remain noteworthy. Short-term programs are generally more acceptable and sustainable for older adults, allowing them to experience positive subjective feedback and psychological reinforcement within a shorter timeframe, which can, in turn, support the continuation of more sustained exercise behaviors (Ashton et al., 2020). Particularly in the initial stage of exercise intervention, short-term programs can serve as an important “initiation phase” strategy, helping older adults gradually develop exercise habits, enhance self-efficacy, and build confidence, thereby laying the groundwork for subsequent medium- and long-term interventions (Nouchi et al., 2012; Arai et al., 2007). Therefore, from a practical perspective, the determination of intervention duration should comprehensively consider older adults' physiological conditions, psychological adaptability, and individual motivational differences, aiming to design progressive, stage-based exercise programs that ensure both effectiveness and long-term adherence.

This study systematically evaluated the effects of aerobic exercise on improving sleep quality among older adults with sleep problems. By comparatively analyzing intervention frequency, session duration, and total intervention period, the study provides insight into factors that may influence intervention effectiveness. However, several limitations should be acknowledged. Although subgroup analyses were conducted, substantial heterogeneity remained, which may be partly attributable to differences in aerobic exercise modalities, sleep assessment tools, and study design. In addition, the literature reviewed was limited to peer-reviewed studies published in English, which may have introduced potential publication and language-related biases. Moreover, a large proportion of the included studies employed Tai Chi–based interventions, and several trials had relatively small sample sizes, which may have influenced the robustness of the pooled estimates. Many of the studies included took place in East Asia; as a result, cultural and contextual variations might pose a limitation, and one should interpret the findings' applicability to other populations with care. Ultimately, this review mainly integrated per-protocol data, which could result in an exaggeration of the actual effect of the intervention; thus, caution is advised when interpreting effect sizes. Future studies with larger sample sizes, more diverse aerobic exercise modalities, and broader geographic representation are warranted.

## Conclusion

The findings of this research suggest that aerobic exercise enhances sleep quality in older adults experiencing sleep issues. Regarding how often interventions are implemented, those carried out three times a week or more tend to produce the most favorable outcomes. Additionally, the findings revealed that sessions lasting 60 min or longer were more effective than shorter ones, while a 12-week intervention period yielded the most favorable clinical benefits. These findings collectively establish a scientific foundation for crafting targeted exercise therapy initiatives and health management approaches focused on enhancing sleep quality among older adults. The research not only validates the beneficial effects of exercise interventions but also highlights the critical need for creating treatment programs that are both efficient and feasible for the senior demographic. When developing exercise prescriptions, it is essential to fully consider individual differences in physical condition, psychological tolerance, and motivation to ensure long-term adherence and safety. From a public health perspective, promoting regular aerobic exercise as a non-pharmacological therapy for improving sleep can reduce reliance on medication, enhance quality of life, and ultimately contribute to the advancement of healthy aging.
