# Supplementary material for: The effects of aerobic exercise on sleep quality in older adults with sleep problems: a systematic review and meta-analysis of randomized controlled trials
Source: Front Psychol. 2026 Feb 24;17:1743800. doi: 10.3389/fpsyg.2026.1743800 (PMC12971442; doi:10.3389/fpsyg.2026.1743800)
Supplement: Supplementary file 1 [file Table_1.docx]

Pubmed

("aerobic exercise*"[Title/Abstract] OR "aerobic training"[Title/Abstract] OR "endurance exercise*"[Title/Abstract] OR "cardiovascular exercise*"[Title/Abstract] OR "swimming"[Title/Abstract] OR "bicycle"[Title/Abstract] OR "walking"[Title/Abstract] OR "running"[Title/Abstract] OR "tai chi"[Title/Abstract]) AND ("Aged"[MeSH Terms] OR "Elderly"[Title/Abstract]) AND ("Sleep"[MeSH Terms] OR "Sleep Quality"[MeSH Terms] OR ("sleep disturbance*"[Title/Abstract] OR "sleep maintenance"[Title/Abstract] OR "sleep disorder*"[Title/Abstract] OR "sleep problem*"[Title/Abstract] OR "sleeplessness"[Title/Abstract] OR "sleep duration*"[Title/Abstract] OR "sleep health"[Title/Abstract])) AND ("randomized controlled trial"[Title/Abstract] OR "randomized"[Title/Abstract] OR "placebo"[Title/Abstract])
