# Supplementary material for: The effects of aerobic exercise on sleep quality in older adults with sleep problems: a systematic review and meta-analysis of randomized controlled trials
Source: Front Psychol. 2026 Feb 24;17:1743800. doi: 10.3389/fpsyg.2026.1743800 (PMC12971442; doi:10.3389/fpsyg.2026.1743800)
Supplement: Supplementary file 2 [file Table_2.docx]

**Supplementary Table S1. GRADE Evidence Profile**

The certainty of evidence for the outcome 'sleep quality' was assessed using the GRADE approach in GRADEpro software (version 3.6, McMaster University, Hamilton, Canada). A total of 12 randomized controlled trials (RCTs) involving 1,008 participants were included. The pooled analysis indicated that the mean sleep quality in the intervention groups was 0.98 standard deviations lower (95% CI: −1.36 to −0.60) compared with the control groups, favoring the exercise intervention.

In the GRADE evaluation:
- Risk of bias: not serious
- Inconsistency: serious (−1)
- Indirectness: not serious
- Imprecision: not serious
- Publication bias: undetected

Accordingly, the overall certainty of evidence was rated as 'MODERATE' (⊕⊕⊕○). The downgrade by one level was due to inconsistency across studies (I² > 50%), likely attributable to differences in intervention types and durations. This finding suggests a moderate level of confidence that exercise interventions can improve sleep quality, although further high-quality RCTs are warranted.

| **Outcome** | **No. of studies (Design)** | **No. of participants** | **Risk of bias** | **Inconsistency** | **Imprecision** | **Certainty (GRADE)** |
| --- | --- | --- | --- | --- | --- | --- |
| Sleep quality | 12 (RCTs) | 1,008 | Not serious | Serious (−1) | Not serious | ⊕⊕⊕○ Moderate |

Footnote:
a. Some included studies lacked blinding or allocation concealment.
b. Considerable heterogeneity across studies (I² > 50%).
c. The quality of evidence was downgraded by one level for inconsistency.
