# Supplementary figures and images for: The effects of aerobic exercise on sleep quality in older adults with sleep problems: a systematic review and meta-analysis of randomized controlled trials
Source: Front Psychol. 2026 Feb 24;17:1743800. doi: 10.3389/fpsyg.2026.1743800 (PMC12971442; doi:10.3389/fpsyg.2026.1743800)

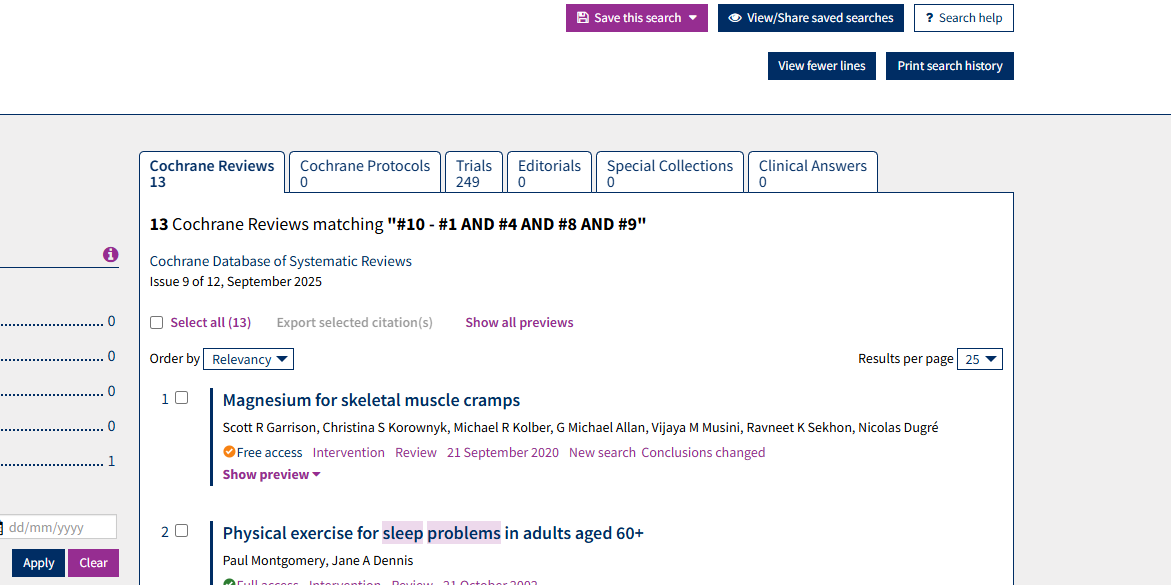

Supplement: Supplementary file 3 [file Image_1.png]

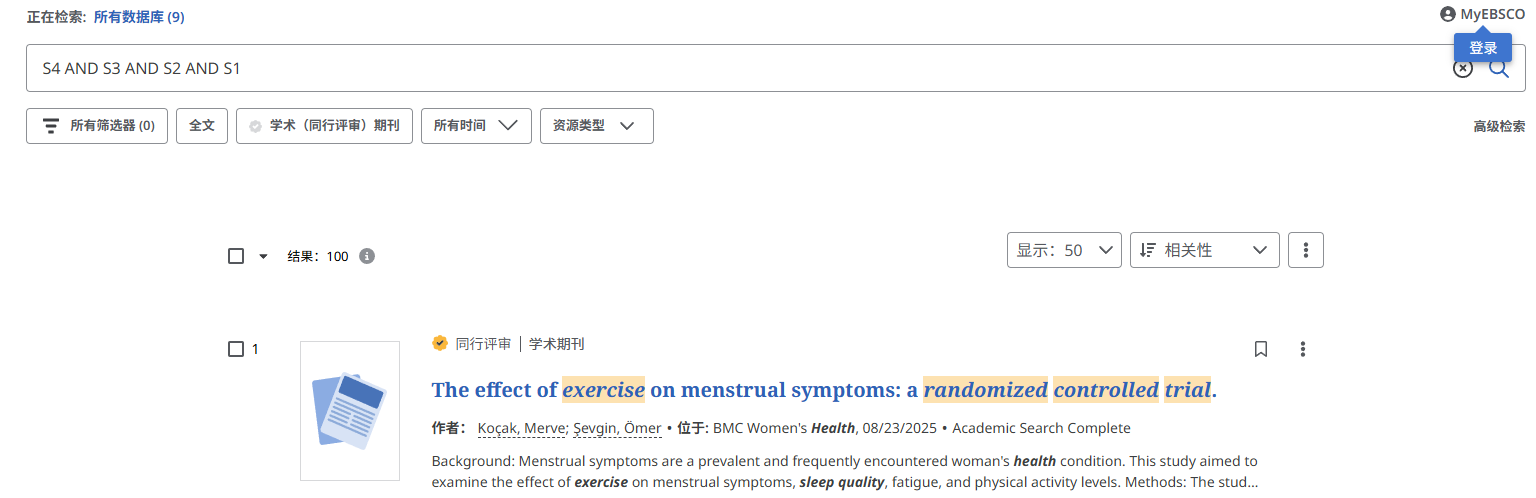

Supplement: Supplementary file 4 [file Image_2.png]

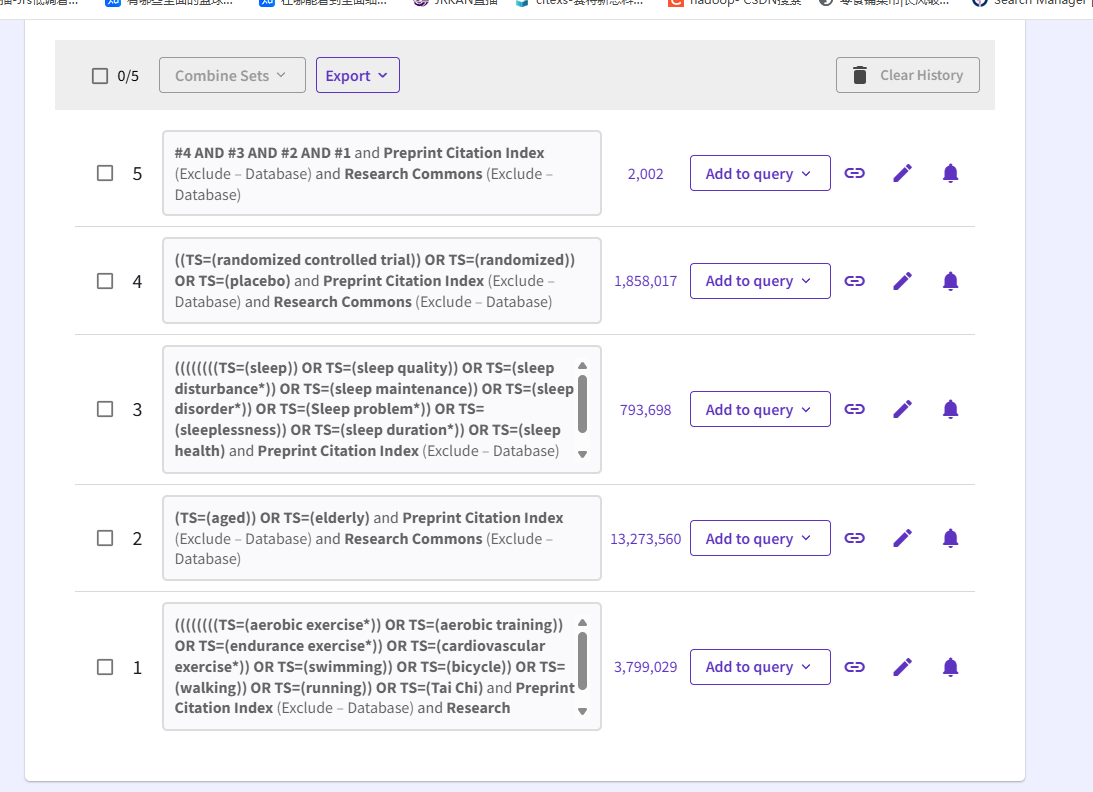

Supplement: Supplementary file 5 [file Image_3.png]

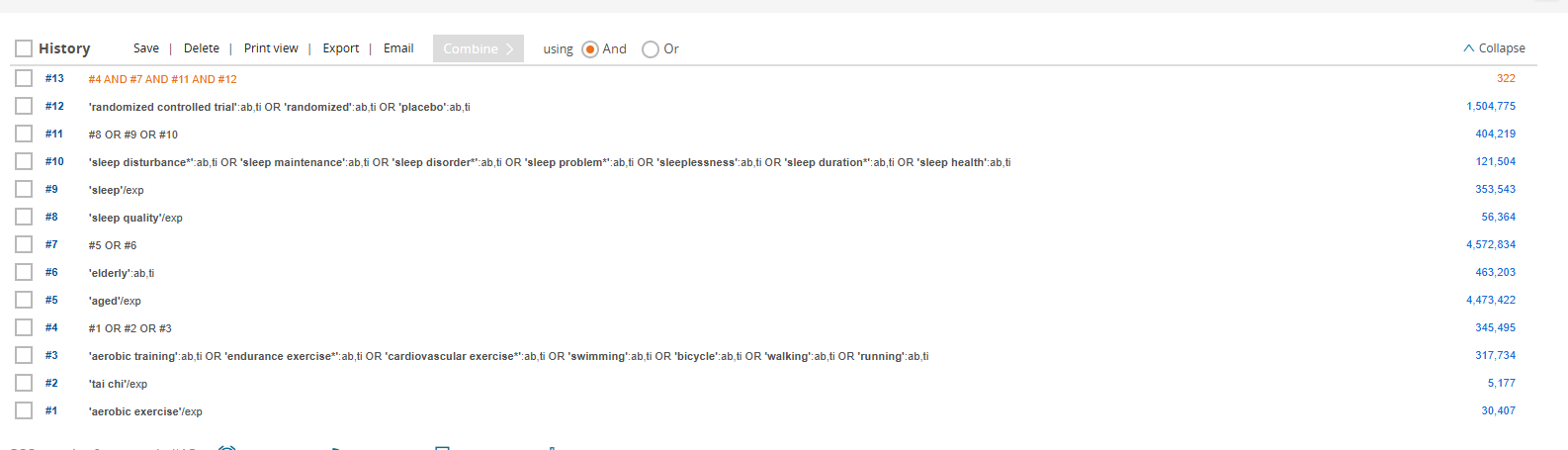

Supplement: Supplementary file 6 [file Image_4.png]

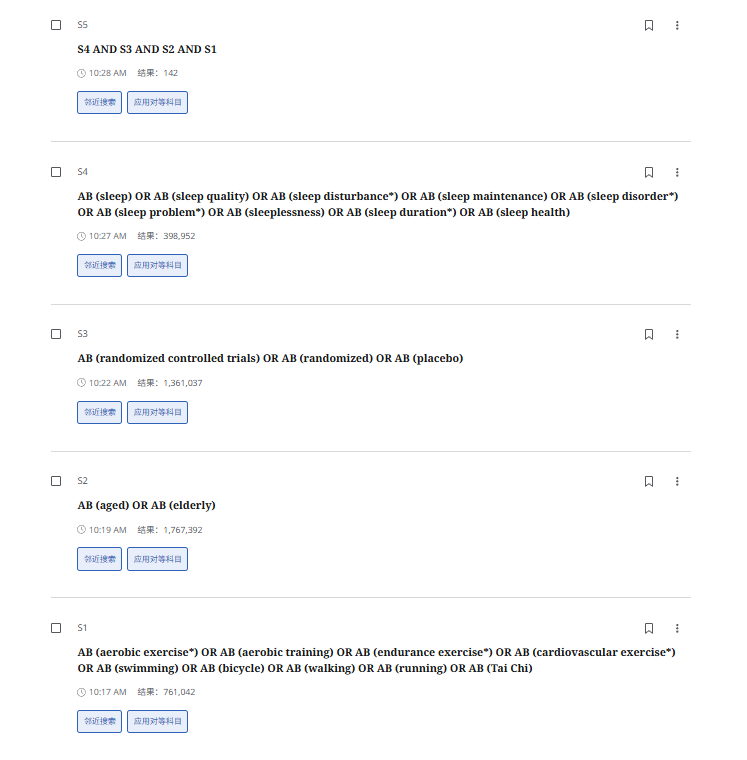

Supplement: Supplementary file 7 [file Image_5.png]

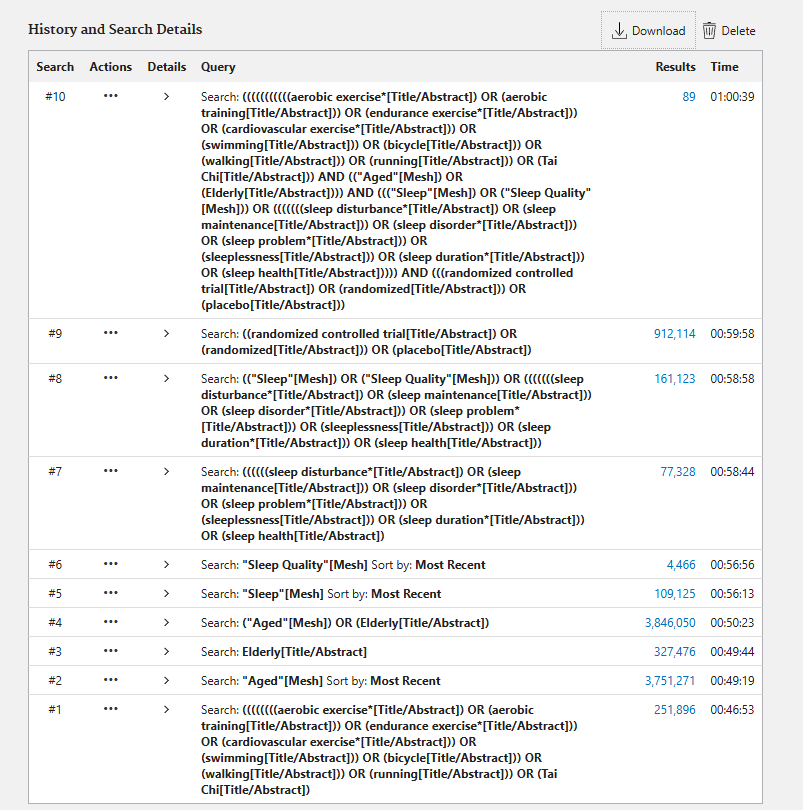

Supplement: Supplementary file 8 [file Image_6.png]

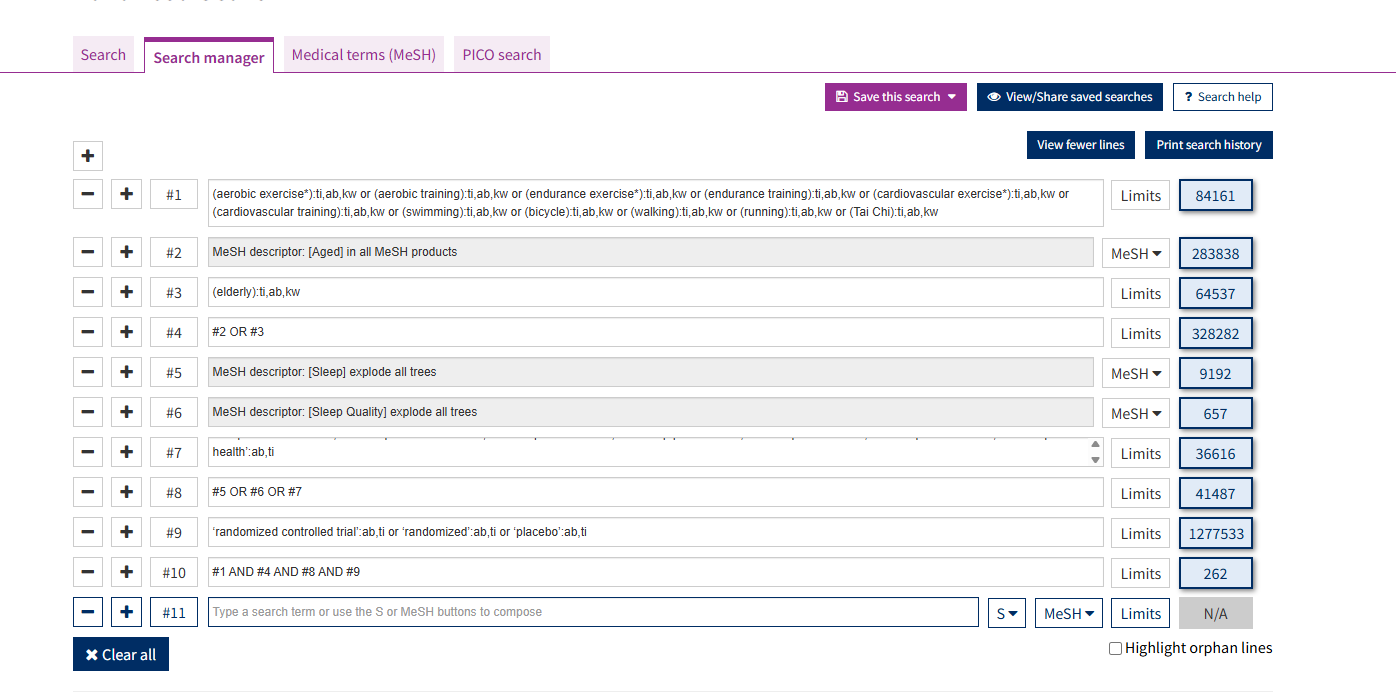

Supplement: Supplementary file 9 [file Image_7.png]
